# Influence of landscape heterogeneity on entomological and parasitological indices of malaria in Kisumu, Western Kenya

**DOI:** 10.1186/s13071-022-05447-9

**Published:** 2022-09-27

**Authors:** Wilfred Ouma Otambo, Patrick O. Onyango, Chloe Wang, Julius Olumeh, Benyl M. Ondeto, Ming-Chieh Lee, Harrysone Atieli, Andrew K. Githeko, James Kazura, Daibin Zhong, Guofa Zhou, John Githure, Collins Ouma, Guiyun Yan

**Affiliations:** 1 Department of Zoology, Maseno University, Kisumu, Kenya; 2 International Centre of Excellence for Malaria Research, Tom Mboya University College-University of California Irvine Joint Lab, Homa Bay, Kenya; 3 Program in Public Health, University of California Irvine, Irvine, CA USA; 4 School of Natural and Environmental Science, Newcastle University, Newcastle, UK; 5 Department of Biology, University of Nairobi, Nairobi, Kenya; 6 Centre for Global Health Research, Kenya Medical Research Institute, Kisumu, Kenya; 7 Department of Pathology, School of Medicine, Case Western Reserve University, Cleveland, OH USA; 8 Centre for Global Health and Diseases, School of Medicine, Case Western Reserve University, Cleveland, OH USA; 9 Department of Biomedical Sciences and Technology, Maseno University, Kisumu, Kenya

**Keywords:** *Anopheles* density, *Plasmodium* infection prevalence, Landscape, Risk factors

## Abstract

**Background:**

Identification and characterization of larval habitats, documentation of *Anopheles* spp. composition and abundance, and *Plasmodium* spp. infection burden are critical components of integrated vector management. The present study aimed to investigate the effect of landscape heterogeneity on entomological and parasitological indices of malaria in western Kenya.

**Methods:**

A cross-sectional entomological and parasitological survey was conducted along an altitudinal transect in three eco-epidemiological zones: lakeshore along the lakeside, hillside, and highland plateau during the wet and dry seasons in 2020 in Kisumu County, Kenya. Larval habitats for *Anopheles* mosquitoes were identified and characterized. Adult mosquitoes were sampled using pyrethrum spray catches (PSC). Finger prick blood samples were taken from residents and examined for malaria parasites by real-time PCR (RT-PCR).

**Results:**

Increased risk of *Plasmodium falciparum* infection was associated with residency in the lakeshore zone, school-age children, rainy season, and no ITNs (*χ*^2^ = 41.201, *df* = 9, *P* < 0.0001). Similarly, lakeshore zone and the rainy season significantly increased *Anopheles* spp. abundance. However, house structures such as wall type and whether the eave spaces were closed or open, as well as the use of ITNs, did not affect *Anopheles* spp. densities in the homes (*χ*^2^ = 38.695, *df* = 7, *P* < 0.0001). *Anopheles funestus* (41.8%) and *An. arabiensis* (29.1%) were the most abundant vectors in all zones. Sporozoite prevalence was 5.6% and 3.2% in the two species respectively. The lakeshore zone had the highest sporozoite prevalence (4.4%, 7/160) and inoculation rates (135.2 infective bites/person/year). High larval densities were significantly associated with lakeshore zone and hillside zones, animal hoof prints and tire truck larval habitats, wetland and pasture land, and the wet season. The larval habitat types differed significantly across the landscape zones and seasonality (*χ*^2^ = 1453.044, *df* = 298, *P* < 0.0001).

**Conclusion:**

The empirical evidence on the impact of landscape heterogeneity and seasonality on vector densities, parasite transmission, and *Plasmodium* infections in humans emphasizes the importance of tailoring specific adaptive environmental management interventions to specific landscape attributes to have a significant impact on transmission reduction.

**Graphical abstract:**

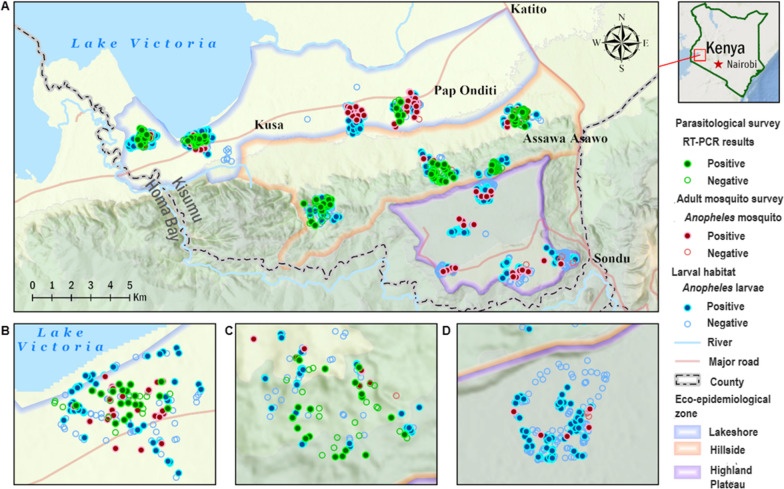

**Supplementary Information:**

The online version contains supplementary material available at 10.1186/s13071-022-05447-9.

## Background

Malaria remains a major public health concern as well as a leading cause of disease and death in Africa. Kenya is currently ramping up malaria control interventions to reduce disease burden and ultimately eliminate the disease [1]. Despite increased interventions, existing control and treatment tools have not sufficiently suppressed *Plasmodium* infection in western Kenya [2]. Approximately 75% of Kenya's 47.5 million people [3] are at risk of malaria, with the Lake Victoria basin having the highest prevalence of infection [1]. Heterogeneity and dynamic changes in the malaria landscape and vector ecology will always pose a challenge to intervention strategies.

Rainfall pattern influences vector occurrence indirectly by determining larval habitat quality and stability [4, 5]. Fluctuations in the wet and dry seasons may change the timing of *Anopheles* mosquito seasonal activity and affect the survival and transmission of malaria vectors as well as the parasites' development rates [4, 6, 7]. As malaria vector and pathogen transmission cycles respond to increasing variability and changes in rainfall pattern, a short transition period between the dry and wet seasons may increase the risk of endemic vector-borne diseases [8, 9].

The environment in which humans live is a strong determinant of *Plasmodium* infection, and the degree of interaction with infectious malaria vectors determines the level of parasite infection [10, 11]. Variation in vector ecology and disease burden across landscape may result in non-homogeneous exposure to transmission risks [12]. In western Kenya's lowlands, which are prone to flooding, and swamps that promote vector breeding and abundance, malaria is common [13–15]. The highlands, on the other hand, are made up of hills and valleys with varying drainage characteristics, resulting in a sparse distribution of larval habitats and a low malaria prevalence [16]. The uneven distribution of larval habitats may have an impact on vector distribution and transmission risks. A shift in vector ecology may alter the vector's host-seeking behavior, influencing malaria epidemiology [14]. Land use for economic activities may result in water pooling, which has the potential to support vector breeding and vector population. Furthermore, the type of household structure, proximity of human settlements to larval habitat, socioeconomic status, and use of insecticide-treated bednets have all been linked to the risk of *Plasmodium* infection [17, 18]. The diverse malaria eco-epidemiological settings and local vector ecologies necessitate interventions that work best for each setting as malaria epidemiology fluctuates over time and correlates with the success of control programs [13]. Understanding the vector ecology and persistence *Plasmodium* infection in Western Kenya across heterogeneous landscape will allow for appropriate target-specific integrated vector management limiting vector breeding and human-vector contact. The current study assessed the role of landscape heterogeneity as defined as topography and seasonality on malaria entomological and parasitological indices along an altitudinal transect of western Kenya's lowland Lake Victoria, hillside, and highland plateau.

## Methods

### Study area and design

**Fig. 1:**
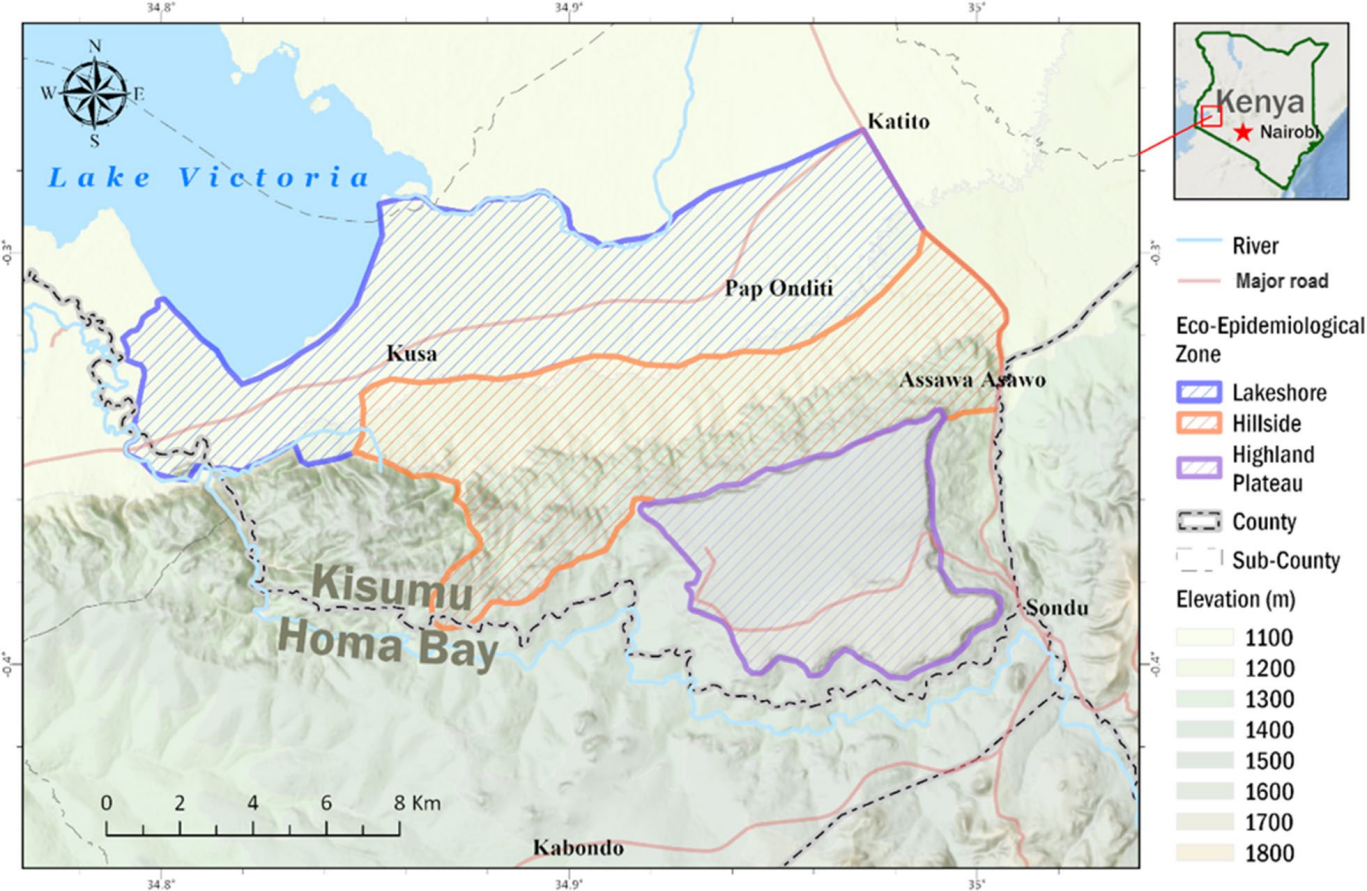
Map of Nyakach Sub-County in Kisumu County showing the study eco-epidemiological zones. Lakeshore zone (highlighted in blue), hillside zone (highlighted in brown), and highland plateau zone (purple highlighted)

The study was carried out in the Nyakach Sub-County of Kisumu County, which is located in western Kenya on the lakeside of Lake Victoria, latitude − 0.33333°S and longitude 34.99100°E. The study area was categorized into three eco-epidemiological zones based on a previous study [12]: lakeshore, hillside, and the highland plateau (Fig. 1). Landscapes in the three zones are very different from each other [12]. The lakeshore consists of flat plain, with common stable larval habitats and an elevation of 1100–1200 m above sea level. The hillside zone located at 1200–1500 m consists of hilly areas with unstable larval habitats and scarce permanent larval habitats. The highland plateau has elevations ranging from 1500 to > 1680 m with high human population density. In all of the study zones, the majority of residents are subsistence farmers. The entomological and parasitological survey was conducted in July (wet season) and November (dry season) of 2020. Each of the three zones had four clusters, with 150 households in each. From each cluster's 150 households, 20 and 25 households were chosen at random for adult mosquito collection and parasitological survey, respectively. The same households were chosen for adult mosquito collection and parasitological surveys during the dry and wet seasons.

### Larval habitat sampling

All potential larval habitats were sampled using a standard dipper (350 ml capacity, BioQuip Products, Inc., Compton, CA, USA) during the dry and wet season. The scooped water was poured in a white plastic tray and carefully examined for *Anopheles* larvae. The sampling took place in the morning (09:00–12:00). The larvae were taxonomically identified using referenced keys, and the *Anopheles* larvae were separated from the Culicine larvae and counted separately [19, 20]. All larvae instars and pupae were sampled, counted, and recorded. The larvae collected were classified as early instars (L1 and L2) or late instars (L3 and L4). The larval density was estimated as the average number of larvae collected per dip. Anopheline larvae were transported to the International Centre of Excellence for Malaria Research (ICEMR) in Homa Bay and reared into adults in the insectary. The larvae were fed TetraMin® fish meal and kept at 27 ± 2 °C. Of all the larvae that survived to adults, further identification was performed using taxonomic keys [21]. No sibling species identification by PCR analysis was performed.

### Characterization of larval habitats

Larval habitats were categorized based on: landscape zones (lakeshore, hillside, highland plateau), larval habitat type (drainage ditch, river edge/reservoir shoreline, swamp, animal hoof print, tire track, manmade pond, natural pond, rock pool, water container, and brick pit), seasonality (dry and wet), land use type (i.e. environment surrounding the larval habitat), vegetation cover, substrate type, proximity to the nearest household, presence of predators and algae in the larval habitats, and larval habitat size as measured by larval habitat length, width, and depth. The habitats were further characterized as: drainage ditch, river edge, swamp, animal hoof print, tire track, manmade pond, natural pond/rain pool, rock pool, water container, and brick pit (Table 1).

### Adult mosquito collection

Adult mosquitos were collected using the pyrethrum spraying catch (PSC) method in 80 selected houses in each zone, for a total of 240 households sampled over 2 days in each dry and wet season. After obtaining consent from the household head, mosquito collection and questionnaire survey were conducted. The PSCs were done between 08:00 am and 12:00 pm [22]. The mosquitoes were collected and stored in 1.5-ml Eppendorf tubes with silica gel desiccant and cotton wool before being transported to ICEMR laboratory in Homa Bay for further analysis. *Anopheles* mosquitoes were identified taxonomically according to Coetzee [21] and females stored on silica gel at room temperature pending further sibling species ID analysis.

Questionnaire survey included the following information on household population size, ITN ownership and use, wall material type, and open vent. ITN use was defined as sleeping under an insecticide-treated net the night before the survey. The wall material type was categorized into three (stone/block/brick, mud and wood, and mud and cement). The availability of open ventilation in the household was classified as open vent.

### Parasitological surveys

A semi-structured questionnaire was administered to the household heads who agreed to participate in the study. The study questionnaire collected information on age, gender, house structure type, and ITN use, as described in the questionnaire surveys. Participants were divided into three age groups based on risk of infection as children < 5 and school-going children and adults (< 5 years old, 5–14 years old, and ≥ 15 years old). To test for *Plasmodium* parasite infections, finger prick dry blood spots (DBS) on filter paper were collected from 100 households in each zone. In each dry and wet season, 300 residents from the lakeshore zone, 285 residents from the hillside zone, and 277 residents from the highland plateau zone were tested for real time-PCR (RT-PCR) diagnosis.

### Molecular identification of mosquito species, blood meal, and sporozoite infections

Adult mosquitoes were cut in half to separate the head and thorax from the abdomen. The Chelex resin (Chelex®-100) method [23] was used to extract mosquito DNA from the head and thorax, while the abdomen was preserved for blood meal analysis. Mosquito species identification was accomplished using multiplex PCR in T100™ Thermal Cycler (Bio-Rad, Hercules, CA, USA) with the primers previously listed [24, 25]. The polymerase chain reaction (PCR) method was used to identify members of the *An. gambiae* complex to the species level and *An. funestus* following protocols developed for *An. gambiae* (s.l.) [24] and *An. funestus* (s.l.) [26, 27]. Identification of blood meals in the fed mosquitoes was performed using the multiplexed PCR-based methods as described by Kent et al. [28]. Analysis of *Plasmodium* sporozoite infection was conducted using multiplexed quantitative PCR (qPCR) assay [29].

### DNA extraction and screening for *Plasmodium* parasite

The Chelex resin (Chelex-100) saponin method was used with minor modifications [30] to extract the genomic DNA from dried blood spots on filter paper. Primers and probes specific to *Plasmodium* species were used to target 18S ribosomal RNA [31] to confirm the presence of parasite DNA on QuantStudio™ 3 Real-Time PCR.

### Data analysis

The IBM SPSS statistical software version 21.0 was used to analyze the data (IBM corp., Armonk, NY). The chi-square test was used to determine whether there was a significant difference in larval habitat types across topographical zones and seasons. The number of *Anopheles* larvae divided by the number of dips taken from each larval habitat was used to calculate *Anopheles* larval density. The Kruskal-Wallis *H* test was used to determine whether there were any significant differences in the composition and abundance of *Anopheles* larvae across different larval habitat types. The negative binomial regression was run to predict *Anopheles* larval density based on landscape zones, seasonality, larval habitat type, surrounding land use type, substrate type, larval habitat size, vegetation cover, shade cover, predator presence, and algae presence. Negative binomial regression was used to determine whether landscape zones, seasonality, household structure type, open eaves, and ITN usage were significant predictors of *Anopheles* vector abundance. The sporozoite infection rate was calculated by dividing the number of mosquitoes positive for *Plasmodium* sporozoites by the total number of mosquitoes analyzed for sporozoite infections [9, 32–34]. The chi-square test was used to compare sporozoite infection among mosquito species. Human blood meal indices (HBI) were calculated by dividing the number of mosquitos that tested positive for human blood meal by the total number of mosquitos analyzed for blood meal [28, 35]. The annual entomological inoculation rate (EIR) of *Anopheles* mosquitoes collected by PSCs was calculated using the following formula: (number of fed mosquitoes caught by PSC/number of human occupants who spent the night in the sprayed house) × (human blood meal indices) × (PSC based sporozoite infection) × 365 [32, 33, 36–38]. Landscape, age group, gender, bednet usage, wall type, and seasonality were all tested as predictors of malaria infections in humans using logistic regression. Significance level was set as *P* ≤ 0.05 for all tests and all regression independent variables.

## Results

### Distribution of larval habitats across topographical zones and seasonality

**Fig. 2:**
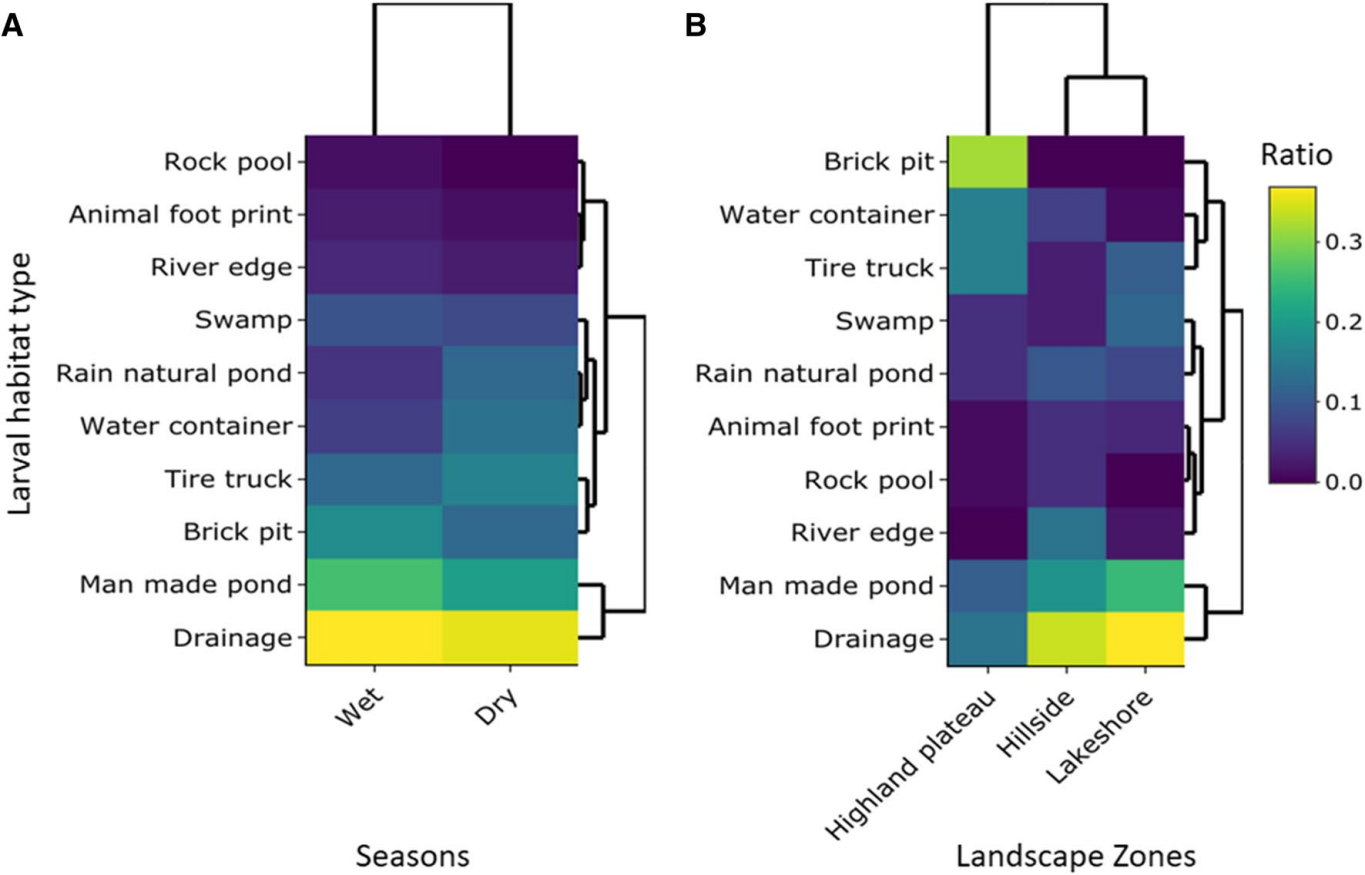
Larval habitat distribution cluster heat map. **A** Larval habitat distribution by seasonality. **B** Larval habitat distribution across topography. The hierarchical clustering dendrogram pattern represents the relationship between larval habitat type and landscape zones or seasons based on average linkage and Euclidean distance. The use of the same color indicates the availability of larval habitat types across landscape zones or seasons

During the study, 10 different types of larval habitats were identified, with a total of 1315 habitats encountered and recorded. The most common were drainage ditches (27.3%), followed by manmade ponds (18.5%), brick pits (12.4%), tire truck (11.2%), water container (8.1%), swamp (7.6%), natural pond (7.1%), river edge (3.7%), animal hoof print (2.7%), and rook pool (1.4%). The ten larval habitat types differed significantly across the topographical zones (Fig. 2, *χ*^2^ = 616.351, *df* = 18, *P* < 0.0001). The lakeshore zone had the most larval habitats (40.6%) of the 1315 identified, followed by the highland plateau zone (38.6%) and then the hillside zone (20.8%) (Fig. 2). The ten larval habitat types differed significantly across seasons (*χ*^2^ = 38.815, *df* = 9, *P* < 0.0001). The wet season had more larval habitats (59.8%) comparatively followed by the dry season (40.2%) (Fig. 2).

### *Anopheles* larval composition and abundance by larval habitat types across topographical zones

There was a significant difference in the composition of *Anopheles* larvae across the habitat types [*F*(39, 8020) = 191.2, *P* < 0.0001]. In total, 2505 *Anopheles* larvae were morphologically identified belonging to four species, which included *An. gambiae* (s.l.), (95.7%, *n* = 2398), *An. funestus* (2.2%, *n* = 56), *An. coustani* (1.8%, *n* = 46), and *An. pharoensis* (0.2%, *n* = 5). Furthermore, drainage ditch had the highest abundance (28.9%) of *Anopheles* larvae (Table 2). The highest proportion of *An. gambiae* (s.l.), *An. funestus,* and *An. coustani,* and *An. pharoensis* were found in the drainage ditch (29.1%), manmade pond (28.6%), and natural pond (30.4%), respectively. *Anopheles pharoensis* was only found in the drainage ditch (Table 2). In the lakeshore zone, the *An. gambiae* (s.l.) (*n* = 1500) proportion differed significantly across the larval habitat types (H_9_ = 63.81, *P* < 0.0001), with manmade ponds (31.3%) having the highest composition. The proportion of *A. funestus* (*n* = 30) was highest in manmade ponds (36.7%); *An. coustani* (*n* = 15) was mainly found in manmade ponds (46.7%). Only the drainage ditch had *An. pharoensis* (Table 2).

**Table 1:** Characterization of larval habitats

Larval habitat survey	Options
1. Study site	Nyakach Sub-County-Kisumu County
2. Eco-epidemiological zone	LK: lakeshore, MD: hillside, NB: highland plateau
3. Habitat serial number	
4. Larval habitat type	A. Drainage ditch: Small to medium depression with water pools formed to channel or drain water runoffs B. River edge: Bodies of water along the river's banks, shores, and edges C. Swamp: Area of low-lying, uncultivated ground with water collects, bogs, or marshes D. Animal hoof print: impressions and depressions on the ground caused by water-filled animal hooves E. Tire track aquatic impressions left by tires on the surface onto which a vehicle drove F.. Manmade pond: Any dug areas filled with water, such as dams, water pans, and fish ponds, among others G. Natural pond: Any depressions filled with rainfall water that had not been dug by humans H. Rock pool: Collections of water in rocks that can support larval breeding I. Water container: any container, pots, or bottles filled with water J: Brick pit: Depressions in the ground caused by brick-making activities
5. Landuse type (surrounding environment)	(1) Forest/shrubland; (2) cultivated land; (3) grassland/pasture; (4) swamp
6. Vegetation coverage %	Based on visual observation, calculated by estimating the percentage of the larval habitat covered
7. Substrate type	(1) Sand/gravel; (2) mud/dirt; (3) plastic/container
8. Distance to nearby house	(1) Less than 100 m; (2) 100–200 m; (3) over 200 m
9. Predators	Each larval habitat assessed for the presence or absence of aquatic predators
10. Algae	The presence or absence of algae visually assessed in the larval habitat
Habitat measure	
11a. Length (m)	Measured and recorded in meters
11b. Width (m)	Measured and recorded in meters
11c. Depth (m)	Measured using a meter stick from various locations and the average depth taken

### Factors associated with *Anopheles* larval density

Negative binomial regression analysis of *Anopheles* larval density illustrated the following risk factors: landscape zones, seasonality, larval habitat type, surrounding land use type, substrate type, larval habitat size, and predation in the larval habitat. However, presence of vegetation cover, larval habitat distance to a nearby household (*P* = 0.206), and water algae were not significant predictors of larval density (*χ*^2^ = 1453.044, *df* = 298, *P* < 0.0001) (Table 3). Compared to the highland plateau, *Anopheles* larval density was 3.23 (95% CI = 2.50–4.18, *P* < 0.0001) times higher in the lakeshore zone and 1.81 (95% CI = 1.32–2.48, *P* < 0.0001) times higher in the hillside zone. *Anopheles* larval density was 4.59 (95% CI = 3.61–5.83, *P* < 0.0001) times higher during the wet season than the dry season (Table 3).

**Table 2:** *Anopheles* larvae composition and abundance in various larval habitat types across landscape zones

*Anopheles* larvae identified		Larval habitat type *n* (%)										Total	Kruskal-Wallis statistics	*P*-value
		Drainage	River edge	Swamp	Animal footprint	Tire track	Manmade pond	Natural pond	Rock pool	Water container	Brick pit			
Lake-shore	*An. gambiae s.l*	448 (29.9)	28 (1.7)	244 (16.3)	82 (5.5)	161 (10.7)	469 (31.3)	68 (4.5)	0	0	0	1500	63.81	< 0.0001
	*An funestus*	7 (23.3)	2 (6.7)	7 (23.3)	0	3 (10.0)	11 (36.7)	0	0	0	0	30	64.49	< 0.0001
	*An. pharoensis*	4 (100)	0	0	0	0	0	0	0	0	0	4	39	< 0.0001
	*An. coustani*	3 (20.0)	0	3 (20.0)	0	0	7 (46.7)	2 (13.3)	0	0	0	15	40.56	< 0.0001
	Total	462 (30.3)	30 (1.9)	254 (16.4)	82 (5.3)	164 (10.6)	487 (30.6)	70 (4.5)	0	0	0	1549	− 413.8	< 0.0001
Hillside	*An. gambiae s.l*	202 (42.1)	45 (9.4)	29 (6.1)	25 (5.2)	17 (3.5)	95 (19.8)	55 (11.5)	9 (1.9)	2 (0.4)	0	479	− 942.9	< 0.0001
	*An funestus*	1 (14.3)	0	1 (14.3)	0	0	5 (71.4)	0	0	0	0	7	35.98	< 0.0001
	*An. pharoensis*	0	0	0	0	0	0	0	0	0	0	0	NA	NA
	*An. coustani*	4 (20.0)	1 (5.0)	0	0	0	4 (20.0)	11 (55.0)	0	0	0	20	69.03	< 0.0001
	Total	207 (40.9)	46 (9.1)	30 (5.9)	25 (4.9)	17 (3.4)	104 (20.6)	66 (13.0)	9 (1.8)	2 (4.0)	0	506	133.44	< 0.0001
Highland plateau	*An. gambiae s.l*	47 (11.2)	0	31 (7.4)	9 (2.1)	12 (2.9)	76 (18.1)	34 (8.1)	2 (4.8)	15 (3.7)	193 (46.1)	419	1046	< 0.0001
	*An funestus*	6 (31.6)	0	0	0	6 (31.6)	0	0	0	0	7 (36.8)	19	55.21	< 0.0001
	*An. pharoensis*	1 (100)	0	0	0	0	0	0	0	0	0	1	NA	NA
	*An. coustani*	0	0	0	0	0	0	1 (9.1)	0	0	10 (90.9)	11	88.98	< 0.0001
	Total	54 (12.0)	0	32 (7.1)	9 (2.0)	18 (4.0)	76 (16.9)	35 (7.8)	2 (0.4)	15 (3.3)	209 (46.4)	450	2100	< 0.0001
Overall	*An. gambiae s.l*	697 (29.1)	73 (3.0)	304 (12.7)	116 (4.8)	190 (7.9)	640 (26.7)	157 (6.5)	11 (0.5)	17 (0.7)	193 (8.0)	2398	580.2	< 0.0001
	*An funestus*	14 (25.0)	2 (3.6)	9 (16.1)	0	9 (16.1)	16 (28.6)	0	0	0	6	56	96.34	< 0.0001
	*An. pharoensis*	5 (100)	0	0	0	0	0	0	0	0	0	5	47.77	< 0.0001
	*An. coustani*	7 (15.2)	1 (2.2)	3 (6.5)	0	0	11(23.9)	14 (30.4)	0	0	10 (21.7)	46	63.73	< 0.0001
	Total	723 (28.9)	76 (3.0)	316 (12.6)	116 (4.6)	199 (7.9)	667 (26.6)	171 (6.8)	11 (0.4)	17 (0.7)	209 (8.3)	2505	− 17.19	< 0.0001

*n*: Number

(%): Proportion

In the lakeshore zone, the mean larval density was 1.61 larvae per dip, with high densities of *Anopheles* larvae collected from animal hoof prints (20.50 larvae per dip). The mean *Anopheles* larval density in the hillside zone was 1.11 larvae per dip, with a high density of *Anopheles* larvae found in animal hoof print larval habitats (8.33 larvae per dip). The mean *Anopheles* larval density in the highland plateau zone was 0.64, with a high density collected from animal hoof prints (9.00 larvae per dip) (Additional file 1: Table S1).

### Adult *Anopheles* species composition and abundance across topographical zones

A total of 221 female *Anopheles* mosquitos were collected by PSC, including *An. gambiae* (s.l.) (*n* = 124), *An. funestus* (*n* = 89), *An. coustani* (*n* = 7), and *An. pharaoensis* (*n* = 1). The mosquito species composition differed significantly between topographical zones (*χ*^2^ = 31.73, *df* = 6, *P* < 0.0001). *Anopheles funestus* (*n* = 89) was the most common primary vector in all the zones followed by *An. arabiensis* (*n* = 62), then *An. gambiae* (s.s.) (*n* = 2). Based on ITS2 (ITS2A/ITS2B primer set) PCR and subsequent sequencing results, 60 specimens were identified to be either *An.* sp. 1 (*n* = 20, GenBank accession number MT408575) or *An.* sp. 9 (*n* = 40, GenBank accession number MT408578) as described by Zhong et al. [29]. *Anopheles funestus* and *An. arabiensis* were the most abundant species in the lakeshore zone, hillside zone, and highland plateau zone (Additional file 1: Table S2).

### *Plasmodium* species sporozoite rates and entomological inoculation rates (EIR)

The overall proportion of *Anopheles* infected with *P. falciparum* sporozoite was 3.8% (*n* = 213). *Anopheles funestus* had the highest sporozoite rate of 5.6% (*n* = 5/89), followed by *An. arabiensis* (3.2%, *n* = 2/62). *Anopheles gambiae* (s.s.) had only two samples with one infection at 50%. Across the landscape zone, the sporozoite rates were higher in the lakeshore zone (4.4%, *n* = 7/160), followed by the highland plateau zone (3.8%, *n* = 1/26) and the hillside zone (3.7, *n* = 1/27) (Additional file 1: Table S2).

The overall human blood meal indices of *An. arabiensis* and *An. funestus* were 63.6% and 30.2%, respectively. The un-amplified mosquito HBI was 34.3%. In the lakeshore zone, the HBIs of *An. arabiensis* and *An. funestus* were 66.7% and 41.7% respectively. In the hillside zone, the HBI of *An. funestus* was 8.3% while there was no blood-fed *An. arabiensis*. In the highland plateau, there was no blood-fed *An. arabiensis* and *An. funestus* though the un-amplified mosquito HBI was 66.7% (Additional file 1: Table S2).

The inoculation rates of *An. gambiae, An. arabiensis*, and *An. funestus* were 26.9, 24.1, and 48.2 infective bites/person/year respectively. The overall inoculation rates were at 20.1 infective bites/person/year. In the lakeshore zone, highland plateau, and hillside zone, inoculation rates were at 135.2, 80.2, and 25.3 infective bites/person/year respectively (Additional file 1: Table S2).

### Predictors of adult *Anopheles* vector abundance

Negative binomial regression revealed that the landscapes of the lakeshore and the rainy season were significant predictors of *Anopheles* adult vector abundance. However, the type of house wall, open eaves, and use of an ITN were not significant predictors of *Anopheles* adult vector abundance (*χ*^2^ = 38.695, *df* = 7, *P* < 0.0001) (Table 4). Compared to the highland plateau, the numbers of adult *Anopheles* vectors were 1.72 (95% CI = 1.02–2.90, *P* = 0.041) times higher in the lakeshore zone while there was no significant difference for the hillside zone (*P* = 0.917). The adult *Anopheles* were 2.17 (95% CI = 1.48–3.20, *P* < 0.0001) times higher during the wet season than the dry season (Table 4).

**Table 3:** Predictive factors associated with *Anopheles* larval densities

Parameter	Details	Coefficient	Odd ratio (95% CI)	*P*-value
Landscape zones	Highland plateau	0	1	
	Lakeshore	1.173	3.23 (2.50–4.18)	< 0.0001
	Hillside	0.594	1.81 (1.32–2.48)	< 0.0001
Habitat type	Brick pit	0	1	
	Drainage	0.055	1.06 (0.74–1.51)	0.761
	River edge	− 0.28	0.76 (0.44–1.30)	0.309
	Swamp	− 0.922	0.40 (0.24–0.66)	< 0.0001
	Animal hoof print	1.731	5.65 (3.48–9.17)	< 0.0001
	Tire truck	0.532	1.70 (1.14–2.55)	0.001
	Manmade pond	− 0.858	0.42 (0.29–0.63)	< 0.0001
	Rain natural pond	− 1.09	0.34 (0.18–0.64)	0.001
	Rock pool	− 1.01	0.36 (0.15–0.88)	0.024
	Water container	− 2.619	0.07 (0.10–0.56)	0.012
Land use type	Cultivated land	0	1	
	Shrub land	0.194	1.21 (0.83–1.78)	0.322
	Pasture/grassland	0.558	1.75 (1.41–2.16)	< 0.0001
	Wetland	0.832	2.30 (1.60–3.29)	< 0.0001
Substrate	Plastic/container	0	1	
	Sand	− 0.0824	0.44 (0.05–3.60)	0.443
	Mad	− 1.338	0.26 (0.03–2.14)	0.211
Distance	> 200 m	0	1	
	< 100 m	− 0.103	0.90 (0.35–2.27)	0.826
	100–200	0.163	1.18 (0.46–3.01)	0.735
Season	Dry	0	1	
	Wet	1.523	4.59 (3.61–5.83)	< 0.0001
Predators	Yes	0	1	
	No	− 0.303	0.77 (0.61–0.89)	0.002
Algae	Yes	0	1	
	No	0.721	2.06 (0.80–5.30)	0.136
Vegetation cover		− 0.001	1.00 (0.99–1.00)	0.451
Habitat size		− 0.008	0.99 (0.99–0.99)	0.034
(Scale)		1		
(Negative binomial)		1		

Dependent variable: *Anopheles* density

Model: (Intercept), topography, habitat type, land use, substrate, distance, season, predator, algae, vegetation cover, habitat size

### Risk factors associated with malaria prevalence

A total of 862 residents were tested for *P. falciparum* infection prevalence (300 in lakeshore, 285 in hillside, and 277 in highland plateau). The prevalence of *P. falciparum* infection was 15.1% (130/862). Increased risk of *P. falciparum* infection was associated with residency in the lakeshore zone, school-age children, rainy season, and no ITNs (*χ*^2^ = 41.201, *df* = 9, *P* < 0.0001. The odds of malaria infection were highest in the lakeshore zone (OR: 3.08, 95% CI = 1.81–5.26, *P* < 0.0001) then hillside (OR: 1.78, 95% CI = 1.00–3.17, *P* = 0.05) zone compared to the highland plateau. The odds of *P. falciparum* infection was higher among the school-going children (OR: 1.92, 95% CI = 1.26–2.92, *P* = 0.002) compared to individuals ≥ 15 years. Not using an ITN was linked to a three-fold increase in the risk of *P. falciparum* infection (OR: 2.84, 95% CI = 1.14–7.09, *P* = 0.025). People who lived in brick or block houses had lower odds of *P. falciparum* infection (OR: 0.58, 95% CI = 0.34–0.98, *P* = 0.040) than those who lived in mud and wood houses. Residents were 1.47 times more likely to contract malaria during the rainy season (OR: 1.47, 95% CI = 1.01–2.13, *P* = 0.044) than during the dry season (Table 5).

**Table 4:** Predictive factors associated with adult *Anopheles* abundance

Parameter	Details	Coefficient	Odd ratio (95% CI)	*P*-value
Landscape zones	Highland plateau	0	1	
	Lakeshore	0.543	1.72 (1.02–2.90)	0.041
	Hillside	− 0.035	1.00 (0.50–1.87)	0.917
Wall type	Mud and cement	0	1	
	Brick/stone	− 0.025	1.00 (0.32–2.96)	0.965
	Mud and wood	0.556	1.74 (1.02–2.98)	0.042
Season	Dry	0	1	
	Wet	0.776	2.17 (1.48–3.20)	< 0.0001
Open vent	Yes	0	1	
	No	0.2	1.22 (0.82–1.82)	0.327
Bed net usage	Use net	0	1	
	No net	− 0.202	0.82 (0.44–1.52)	0.525
(Scale)		1		
(Negative binomial)		1		

Dependent variable: adult *Anopheles* number

Model: (intercept), topography, wall type, season, open vent, bed net usage

**Table 5:** Risk factors associated with* Plasmodium falciparum* infection prevalence

Determinants	Category	Infection *n* (%)	Odd ratio (95% CI)	*P*-value
Landscape zones	Highland plateau	25 (9.0)	1	
	Lakeshore	65 (21.7)	3.08 (1.81, 5.26)	< 0.0001
	Hillside	40 (14.0)	1.78 (1.00, 3.17)	0.05
Age group	≥ 15 years	57 (12.1)	1	
	0–4 years	14 (12.1)	0.95 (0.50, 1.82)	0.871
	5–14 years	59 (21.5)	1.92 (1.26, 2.92)	0.002
Gender	Male	56 (16.9)	1	
	Female	74 (14.0)	0.90 (0.60, 1.34)	0.597
Bed net usage	Use net	123 (14.8)	1	
	No net	7 (23.3)	2.84 (1.14, 7.09)	0.025
Wall type	Mud and wood	94 (17.1)	1	
	Brick and block	19 (10.0)	0.58 (0.34, 0.98)	0.040
	Mud and cement	17 (12.9)	0.72 (0.41, 1.25)	0.244
Seasonality	Dry	61 (12.8)	1	
	Wet	69 (17.8)	1.47 (1.01, 2.13)	0.044

Dependent variable: RT-PCR results

**Fig. 3:**
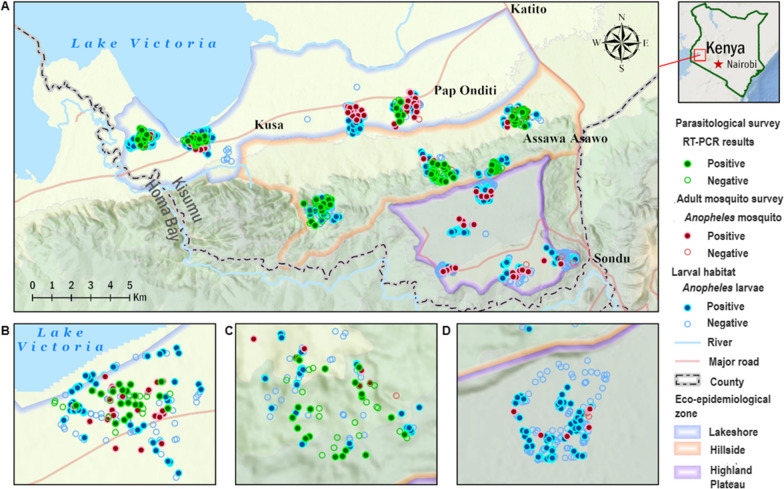
Distribution of larval habitats, households with the adult vector survey, and parasitological survey households. **A** Overview of the study area, including all three eco-epidemiological zones. **B**, **C**, and **D** Distributions of three surveys in lakeshore zone, hillside zone, and highland plateau zone, respectively

Figure 3 depicts the distribution of larval habitats, *Anopheles* adult survey households, and parasitological survey households. The positive in blue dots in the larval habitat survey refers to *Anopheles* larvae collected from those habitats. The positives in red points in the adult vector survey indicate that *Anopheles* mosquitos were collected from that household. The households with green color in the parasitological survey indicate that the resident's RT-PCR result is positive for *Plasmodium* infection.

## Discussion

Landscape, habitat type, land use type, substrate type, distance to a nearby household, seasonality, and predation all predicted high *Anopheles* larval density. In the current study, *An. funestus* and *An. arabiensis* were the most abundant primary vectors in all zones. The primary determinants of mosquito relative abundance were landscape zones and seasonality. Furthermore, lakeshore zone residency, school-aged children, no ITN, living in mud and wood houses, and transmission due to wet season exposure were all associated with a high prevalence of malaria.

The variation in *Plasmodium* infection across the landscape could be attributed to the spatial distribution of larval habitats and the abundance of *Anopheles* vectors. Manmade ponds, drainage ditches, and swamps were the primary larval habitats for *Anopheles* mosquitoes along the lowland lakeshore zone. The findings of the study are consistent with those of a study conducted in western Kenya, where low-gradient landscape zones characterized by broad valley bottoms had a high malaria risk, as opposed to a steep gradient landscape with seasonal variations [39]. Despite the fact that numerous streams cause efficient drainage and diffuse hydrology, resulting in unstable larval habitats, the composition of *Anopheles* larval species and vector abundance remains high in the highland zones. Land use activities such as pond construction, dam construction for water reservoirs, and brick-making leave water-filled depressions on the ground. The water-filled depressions may serve as potential larval habitats for *Anopheles* mosquitoes, which could be one of the factors contributing to the vector population's persistence and malaria infection in the hillside zone. The current study findings are consistent with previous Ethiopian studies that linked environmental modifications for irrigation development activities to proliferation of suitable mosquito larval habitats [40].

*Anopheles* larval density was found to be significantly related to larval habitat type, land use type surrounding the larval habitat, seasonality, and predation. The findings of the current study are consistent with those of a study conducted in western Kenya's Kombewa and Bungoma, which correlated *Anopheles* larval densities to larval habitat type and land use type [41]. The distribution and abundance of *Anopheles* larvae are influenced by hydrological processes that govern the formation and stability of various larval habitat types.

Aquatic predators are well known for influencing the abundance of mosquito larvae in larval habitats and are beneficial biological control agents of mosquito larvae [42]. The current study, however, found that larval densities were lower in larval habitats without predators. This may be due to the presence of other prey in larval habitats, as alternative prey in larval habitats may interfere with the predator larval consumption ability [41]. Other biotic/abiotic factors, such as the presence of green algae, soil substrates, and so on, may, on the other hand, influence larval density. Polluted environments may be detrimental to *Anopheles* larval survival [43].

An increase in mosquito larval habitats leads to an increase in vector density, which eventually leads to an increase in malaria transmission, as has been observed elsewhere [10, 33, 42, 44]. Such findings are consistent with current research findings that show an increase in malaria prevalence and transmission risk in lowland areas due to higher vector abundance than in highland areas. The entomological inoculation rates also revealed that *An. funestus* (48.2 bites/person/year) was a more efficient vector than *An. arabiensis* (24.1 bites/person/year). The EIRs are influenced by topography [45], and the current study found that the EIR was higher in lowland lakeshores (135.2 bites/person/year) than in highland plateaus (80.2 bites/person/year). The finding may be related to a higher risk of malaria infection in lowland areas versus highlands. These findings are backed up in part by the annual EIRs, which revealed significant differences between study sites.

Land use type surrounding the larval habitat was significantly associated with *Anopheles* larval density, with cultivated land negatively influencing larval density. The low density of larvae along cultivated land could be attributed to agricultural insecticides, which may interfere with the *Anopheles* species composition. During the rainy season, the reported productivity of larval habitats correlates with increased vector density and species richness [7, 9, 39]. Intense rainfall, on the other hand, may interfere with mosquito larvae densities due to the flushing of larvae from larval habitats, even though the proportion of *Anopheles* mosquito larvae may increase during and immediately after the rainy season [46].

School-aged children were more likely to contract *P. falciparum* infection in the current study. It has been reported that school-aged children act as reservoirs for infectious gametocytes, spreading infection throughout the community [47–50]. The current study discovered that houses made of mud increased the risk of infection and the abundance of mosquitoes as reported in other studies [13, 51, 52].

According to the current study, larval habitats are confined to the valley bottom, with high intensity of infection in lowland areas and few larval habitats in the highland plateau. This has resulted in a heterogeneous distribution of the vector and parasite burden, which has been documented [10, 39, 53]. This eco-epidemiological variation has implications for vector control programs, as interventions that work well in one setting may not work well in another. Continuously integrating larval source management with LLINs and IRS will reduce the vector population, resulting in a reduction in disease burden. The current study had a limitation in that no sibling species identification by PCR analysis was performed for the *Anopheles* larvae identification.

## Conclusion

The risk of *P. falciparum* infection was linked to residency in the lakeshore zone, school-going age, living in mud houses, rainy season, and not using ITNs. Adult *Anopheles* abundance, on the other hand, was linked to landscape zones and seasonality. Furthermore, high larval densities were predicted by landscape of the lakeshore and hillside zones, animal hoof prints, and tire truck larval habitats, wetland and pasture land, as well as the wet season. The highest abundances of *Anopheles* larvae were found in drainage ditches and manmade ponds. *Anopheles funestus* and *An. arabiensis* were the most abundant adult vectors across the study zone landscape. Understanding the larval habitats of malaria vectors and reducing their availability is critical for malaria control and elimination through the implementation of target specific environmental management interventions, which can significantly reduce malaria burden.

## Supplementary Information

**Additional file 1: Table S1.** *Anopheles* larvae density in various larval habitat types across topography. **Table S2.** *Anopheles* species composition and sporozoite rates, human blood meal index, and entomological inoculation rates (EIR).

## Data Availability

The ITS2 sequences obtained in the study are available in GenBank with accession numbers: MT408575 and MT408578.

## Abbreviations

CI: Confidence interval
DNA: Deoxyribonucleic acid
EIR: Entomological inoculation rate
ICEMR: International Centre of Excellence for Malaria Research
ITN: Insecticide-treated nets
PCR: Polymerase chain reaction
PSC: Pyrethrum spray catches

## References

[1] National Malaria Control Programme, Ministry of Health. The Kenya Malaria Strategy 2019–2023. Nairobi; 2019.

[2] Otambo WO, Olumeh JO, Ochwedo KO, Magomere EO, Debrah I, Ouma C (2022). Health care provider practices in diagnosis and treatment of malaria in rural communities in Kisumu County, Kenya. Malar J.

[3] Kenya National Bureau of Statistics. Kenya population and housing census. Volume 1: population by county and sub-county. 2019 Kenya population and housing census, I(November), 49. https://www.knbs.or.ke/?wpdmpro=2019-kenya-population-and-housing-census-volume-i-population-by-county-and-sub-county.

[4] Dabaro D, Birhanu Z, Negash A, Hawaria D, Yewhalaw D (2021). Effects of rainfall, temperature and topography on malaria incidence in elimination targeted district of Ethiopia. Malar J.

[5] Matsushita N, Kim Y, Ng CFS, Moriyama M, Igarashi T, Yamamoto K (2019). Differences of rainfall–malaria associations in lowland and highland in western Kenya. Int J Environ Res Public Health.

[6] Reiner RC, Geary M, Atkinson PM, Smith DL, Gething PW (2015). Seasonality of *Plasmodium falciparum* transmission: a systematic review. Malar J.

[7] Selvaraj P, Wenger EA, Gerardin J (2018). Seasonality and heterogeneity of malaria transmission determine success of interventions in high-endemic settings: a modeling study. BMC Infect Dis.

[8] Bouvier P, Rougemont A, Breslow N, Doumbo O, Delley V, Dicko A (1997). Seasonality and malaria in a West African village: does high parasite density predict fever incidence?. Am J Epidemiol.

[9] Soma DD, Poda SB, Hien AS, Namountougou M, Sangaré I, Sawadogo JME (2021). Malaria vectors diversity, insecticide resistance and transmission during the rainy season in peri-urban villages of south-western Burkina Faso. Malar J.

[10] Githeko AK, Ayisi JM, Odada PK, Atieli FK, Ndenga BA, Githure JI (2006). Topography and malaria transmission heterogeneity in western Kenya highlands : prospects for focal vector control. Malar J.

[11] Afrane YA, Zhou G, Githeko AK, Yan G (2014). Clinical malaria case definition and malaria attributable fraction in the highlands of western Kenya. Malar J.

[12] Otambo WO, Omondi CJ, Ochwedo KO, Onyango O, Atieli H, Ming-chieh Lee CW (2022). Risk associations of submicroscopic malaria infection in lakeshore, plateau and highland areas of Kisumu County in western Kenya. PLoS ONE.

[13] Mutero CM, Okoyo C, Girma M, Mwangangi J, Kibe L, Ng’ang’a P (2020). Evaluating the impact of larviciding with Bti and community education and mobilization as supplementary integrated vector management interventions for malaria control in Kenya and Ethiopia. Malar J.

[14] Mwakalinga VM, Sartorius BKD, Limwagu AJ, Mlacha YP, Msellemu DF, Chaki PP (2018). Topographic mapping of the interfaces between human and aquatic mosquito habitats to enable barrier targeting of interventions against malaria vectors. R Soc Open Sci.

[15] Ng’ang’a N, Mutunga J, Oliech G, Mutero CM (2019). Community knowledge and perceptions on malaria prevention and house screening in Nyabondo, Western Kenya. BMC Public Health.

[16] Wanjala CL, Githeko AK, Waitumbi JN (2010). Assessing the impact of topography on malaria exposure and malaria epidemic sensitivity in the Western Kenya highlands. Malar J.

[17] Guerra M, De SB, Mabale NN, Berzosa P, Arez AP (2018). Malaria determining risk factors at the household level in two rural villages of mainland Equatorial Guinea. Malar J.

[18] Bannister-tyrrell M, Srun S, Sluydts V, Gryseels C, Mean V, Kim S (2018). Importance of household-level risk factors in explaining micro- epidemiology of asymptomatic malaria infections in Ratanakiri Province, Cambodia. Sci Rep.

[19] Coetzee M (2020). Key to the females of Afrotropical Anopheles mosquitoes (Diptera : Culicidae ). Malar J.

[20] Gillies MT, Coetzee M. A supplement to the Anophelinae of Africa South of the Sahara (Afrotropical Region). (publication no. 55) Johannesburg: South Africa Institute for Medical Research; 1987.

[21] Coetzee M, Craig M, le Sueur D, Coetzee M, Craig M, le Sueur D (2000). Distribution of African malaria mosquitoes belonging to the Anopheles gambiae complex. Parasitol Today.

[22] Silver JB (2007). Mosquito ecology: field sampling methods.

[23] Musapa M, Kumwenda T, Mkulama M, Chishimba S, Norris DE, Thuma PE, Mharakurwa S (2013). A simple Chelex protocol for DNA extraction from Anopheles spp. J Vis Exp.

[24] Scott JA, Brogdon WG, Collins FH (1993). Identification of single specimens of the Anopheles gambiae complex by the polymerase chain reaction. Am J Trop Med Hyg..

[25] Cohuet A, Simard F, Toto JC, Kengne P, Coetzee M, Fontenille D (2003). Species identification within the Anopheles funestus group of malaria vectors in Cameroon and evidence for a new species. Am J Trop Med Hyg.

[26] Koekemoer LL, Lochouarn L, Hunt RH, Coetzee M. Single-strand conformation polymorphism analysis for identification of four members of the Anopheles funestus (Diptera: Culicidae) group. J Med Entomol. 1999;36:125–130. 10.1093/jmedent/36.2.125 10083746

[27] Koekemoer LL, Kamau L, Hunt RH, Coetzee M (2002). A cocktail polymerase chain reaction assay to identify members of the Anopheles Funestus (Diptera: Culicidae) group. Am J Trop Med Hyg.

[28] Kent RJ, Norris DE, Feinstone WH (2005). Identification of mammalian blood meals in mosquitoes by a multiplexed polymerase chain reaction targeting cytochrome B. Am J Trop Med Hyg.

[29] Zhong D, Hemming-Schroeder E, Wang X, Kibret S, Zhou G, Atieli H (2020). extensive new Anopheles cryptic species involved in human malaria transmission in western Kenya. Sci Rep.

[30] Plowe CV, Djimde A, Bouare M, Doumbo O, Wellems TE (1995). Pyrimethamine and proguanil resistance-conferring mutations in *Plasmodium falciparum* dihydrofolate reductase: polymerase chain reaction methods for surveillance in Africa. Am J Trop Med Hyg.

[31] Veron V, Stephane S, Bernard C (2009). Experimental Parasitology Multiplex real-time PCR detection of P. falciparum, P. vivax and P. malariae in human blood samples. Exp Parasitol..

[32] Kilama M, Smith DL, Hutchinson R, Kigozi R, Yeka A, Lavoy G (2014). Estimating the annual entomological inoculation rate for *Plasmodium falciparum* transmitted by Anopheles gambiae s.l. using three sampling methods in three sites in Uganda. Malar J.

[33] Mzilahowa T, Hastings IM, Molyneux ME, McCall PJ (2012). Entomological indices of malaria transmission in Chikhwawa district, Southern Malawi. Malar J.

[34] Tanser FC, Sharp B, Le Sueur D (2003). Potential effect of climate change on malaria transmission in Africa. Lancet.

[35] Garrett-Jones C (1964). The human blood index of malaria vectors in relation to epidemiological assessment. Bull World Health Organ.

[36] Degefa T, Yewhalaw D, Zhou G, Lee MC, Atieli H, Githeko AK (2017). Indoor and outdoor malaria vector surveillance in western Kenya: Implications for better understanding of residual transmission. Malar J.

[37] Khagayi S, Desai M, Amek N, Were V, Onyango ED, Odero C (2019). Modelling the relationship between malaria prevalence as a measure of transmission and mortality across age groups. Malar J.

[38] Shaukat AM, Breman JG, McKenzie FE (2010). Using the entomological inoculation rate to assess the impact of vector control on malaria parasite transmission and elimination. Malar J.

[39] Atieli HE, Zhou G, Lee MC, Kweka EJ, Afrane Y, Mwanzo I (2011). Topography as a modifier of breeding habitats and concurrent vulnerability to malaria risk in the western Kenya highlands. Parasit Vectors.

[40] Hawaria D, Demissew A, Kibret S, Lee MC, Yewhalaw D, Yan G (2020). Effects of environmental modification on the diversity and positivity of anopheline mosquito aquatic habitats at Arjo-Dedessa irrigation development site, Southwest Ethiopia. Infect Dis Poverty.

[41] Debrah I, Afrane YA, Amoah LE, Ochwedo KO, Mukabana WR, Zhong D (2021). Larval ecology and bionomics of Anopheles funestus in highland and lowland sites in western Kenya. PLoS ONE.

[42] Hinne IA, Attah SK, Mensah BA, Forson AO, Afrane YA (2021). Larval habitat diversity and Anopheles mosquito species distribution in different ecological zones in Ghana. Parasit Vectors.

[43] Getachew D, Balkew M, Tekie H (2020). Anopheles larval species composition and characterization of breeding habitats in two localities in the Ghibe River Basin, southwestern Ethiopia. Malar J.

[44] Omalu ICJ, Olayemi IK, Otuu C, Hassan SC, Eke SS, Paul S (2015). Entomological and parasitological indices of malaria transmission in Minna, entomological and parasitological indices of malaria transmission in Minna, Niger State, North Central. Adv Res.

[45] Omukunda E, Githeko A, Ndong’a MF, Mushinzimana E, Atieli H, Wamae P (2013). Malaria vector population dynamics in highland and lowland regions of western Kenya. J Vector Borne Dis.

[46] McCann RS, Messina JP, MacFarlane DW, Bayoh MN, Vulule JM, Gimnig JE (2014). Modeling larval malaria vector habitat locations using landscape features and cumulative precipitation measures. Int J Health Geogr.

[47] Coalson JE, Cohee LM, Buchwald AG, Nyambalo A, Kubale J, Seydel KB (2018). Simulation models predict that school-age children are responsible for most human-to-mosquito *Plasmodium falciparum* transmission in southern Malawi. Malar J.

[48] Whittaker C, Slater HC, Bousema T, Drakeley C, Ghani A, Okell LC. Global patterns of submicroscopic Plasmodium falciparum malaria infection: insights from a systematic review and meta-analysis of population surveys. Lancent Microbe. 2021;2:e366–74. 10.1016/S2666-5247(21)00055-0 PMC8332195 34382027

[49] Walldorf JA, Cohee LM, Coalson JE, Bauleni A, Nkanaunena K, Kapito-Tembo A (2015). School-age children are a reservoir of malaria infection in Malawi. PLoS ONE.

[50] Zhiyong Z, Mitchell RM, Kariuki S, Odero C, Otieno P, Otieno K (2016). Assessment of submicroscopic infections and gametocyte carriage of *Plasmodium falciparum* during peak malaria transmission season in a community-based cross-sectional survey in western Kenya, 2012. Malar J.

[51] Essendi WM, Vardo-Zalik AM, Lo E, Machani MG, Zhou G, Githeko AK (2019). Epidemiological risk factors for clinical malaria infection in the highlands of Western Kenya. Malar J.

[52] Ng'ang'a PN, Okoyo C, Mbogo C, Mutero CM. Evaluating effectiveness of screening house eaves as a potential intervention for reducing indoor vector densities and malaria prevalence in Nyabondo, western Kenya. Malar J. 2020;19:341. 10.1186/s12936-020-03413-3. 10.1186/s12936-020-03413-3 PMC7501660 32950061

[53] Nmor JC, Sunahara T, Goto K, Futami K, Sonye G, Akweywa P (2013). Topographic models for predicting malaria vector breeding habitats: potential tools for vector control managers. Parasites Vectors..

