# Supplementary material for: Influence of landscape heterogeneity on entomological and parasitological indices of malaria in Kisumu, Western Kenya
Source: Parasit Vectors. 2022 Sep 27;15:340. doi: 10.1186/s13071-022-05447-9 (PMC9516797; doi:10.1186/s13071-022-05447-9)
Supplement: Supplementary file 1 — Additional file 1: Table S1. Anopheles larvae density in various larval habitat types across topography. Table S2. Anopheles species composition and sporozoite rates, human blood meal index, and entomological inoculation rates (EIR). [file 13071_2022_5447_MOESM1_ESM.docx]

**Additional file 1: Table S1. Anopheles larvae density in various habitat types across topographical zones**

| **Parameters** | | **Drainage** | **River edge** | **Swamp** | **Animal footprint** | **Tire track** | **Man**  **made pond** | **Natural pond** | **Rock pool** | **Water container** | **Brick pit** | **Total** |
| --- | --- | --- | --- | --- | --- | --- | --- | --- | --- | --- | --- | --- |
| Lakeshore | No. of dips | 197 | 12 | 182 | 4 | 39 | 398 | 125 | 0 | 4 | 2 | 963 |
|  | larvae count | 462 | 30 | 254 | 82 | 164 | 487 | 70 | 0 | 0 | 0 | 1549 |
|  | Larvae/dip(mean) | 2.35 | 2.50 | 1.40 | 20.50 | 4.21 | 1.22 | 0.56 | 0.00 | 0.00 | 0.00 | 1.61 |
| Hillside | No. of dips | 93 | 37 | 24 | 3 | 3 | 170 | 89 | 15 | 21 | 0 | 455 |
|  | Larval count | 207 | 46 | 30 | 25 | 17 | 104 | 66 | 9 | 2 | 0 | 506 |
|  | Larvae/dip(mean) | 2.23 | 1.24 | 1.25 | 8.33 | 5.67 | 0.61 | 0.74 | 0.60 | 0.10 | 0.00 | 1.11 |
| Plateau | No. of dips | 75 | 0 | 71 | 1 | 43 | 137 | 57 | 3 | 83 | 238 | 708 |
|  | Larval count | 50 | 0 | 33 | 9 | 14 | 76 | 40 | 2 | 15 | 211 | 450 |
|  | Larvae/dip(mean) | 0.67 | 0.00 | 0.46 | 9.00 | 0.33 | 0.55 | 0.70 | 0.67 | 0.18 | 0.89 | 0.64 |
| Overall | No. of dips | 365 | 49 | 277 | 8 | 85 | 705 | 271 | 18 | 108 | 240 | 2126 |
|  | Larval count | 719 | 76 | 317 | 116 | 195 | 667 | 176 | 11 | 17 | 211 | 2505 |
|  | Larvae/dip(mean) | 1.97 | 1.55 | 1.14 | 14.50 | 2.29 | 0.95 | 0.65 | 0.61 | 0.16 | 0.88 | 1.18 |

**Additional file 1: Table S2. Anopheles species composition and sporozoites rate, Human blood meal index and Entomological inoculation rates (EIR)**

| **Zones** | **Details** | ***An. gambiae***  **n (%)** | ***An. arabiensis* n (%)** | ***An. funestus* n (%)** | **Un-amplified**  **n (%)** | **Total** |
| --- | --- | --- | --- | --- | --- | --- |
| lakeshore | *Anopheles* Number | 1 (0.6) | 57 (35.6) | 63 (39.4) | 39 (24.4) | 160 |
|  | Sporozoites Infection | 1 (100) | 2 (3.5) | 4 (6.3) | 0 | 7 (4.4) |
|  | Blood meal index | 0 | 66.7 | 41.7 | 18.2 | 41.8 |
|  | EIR | 0 | 45.6 | 88.0 | 0 | 135.2 |
| Hillside | *Anopheles* Number | 0 | 3 (11.1) | 18 (66.7) | 6 (22.2) | 27 |
|  | Sporozoites Infection | 0 | 0 | 1 (5.6) | 0 | 1(3.7) |
|  | Blood meal index | 0 | 0 | 8.3 | 50 | 17.6 |
|  | EIR | 0 | 0 | 12.3 | 0 | 25.3 |
| Highland plateau | *Anopheles* Number | 1 (3.8) | 2 (7.7) | 8 (30.8) | 15 (57.7) | 26 |
|  | Sporozoites Infection | 0 | 0 | 0 | 1(6.7) | 1(3.8) |
|  | Blood meal index | 100 | 0 | 0 | 66.7 | 46.7 |
|  | EIR | 0 | 0 | 0 | 121.3 | 80.2 |
| Totals | *Anopheles* Number | 2 (0.9) | 62 (29.1) | 89 (41.8) | 60 (28.2) | 213 |
|  | Sporozoites Infection | 1(50) | 2 (3.2) | 5 (5.6) | 1(1.7) | 9(4.2) |
|  | Blood meal index | 100 | 63.6 | 30.2 | 34.3 | 38.7 |
|  | EIR | 26.9 | 24.1 | 48.2 | 11 | 20.1 |
